# Supplementary material for: The open-ocean missing backscattering is in the structural complexity of particles
Source: Nat Commun. 2018 Dec 21;9:5439. doi: 10.1038/s41467-018-07814-6 (PMC6303329; doi:10.1038/s41467-018-07814-6)
Supplement: Supplementary file 1 — Supplementary Information [file 41467_2018_7814_MOESM1_ESM.pdf]

SUPPLEMENTARY MATERIAL

The open-ocean missing backscattering is in the structural complexity of particles

Organelli et al.

correspondence to: emanuele.organelli@obs-vlfr.fr (E. Organelli)

Table of Contents

Section 1: Supplementary Figures 1-14 (pages 2-15)
Section 2: Supplementary Table 1 (page 16)
Section 3: Supplementary References (page 17)

Section 1

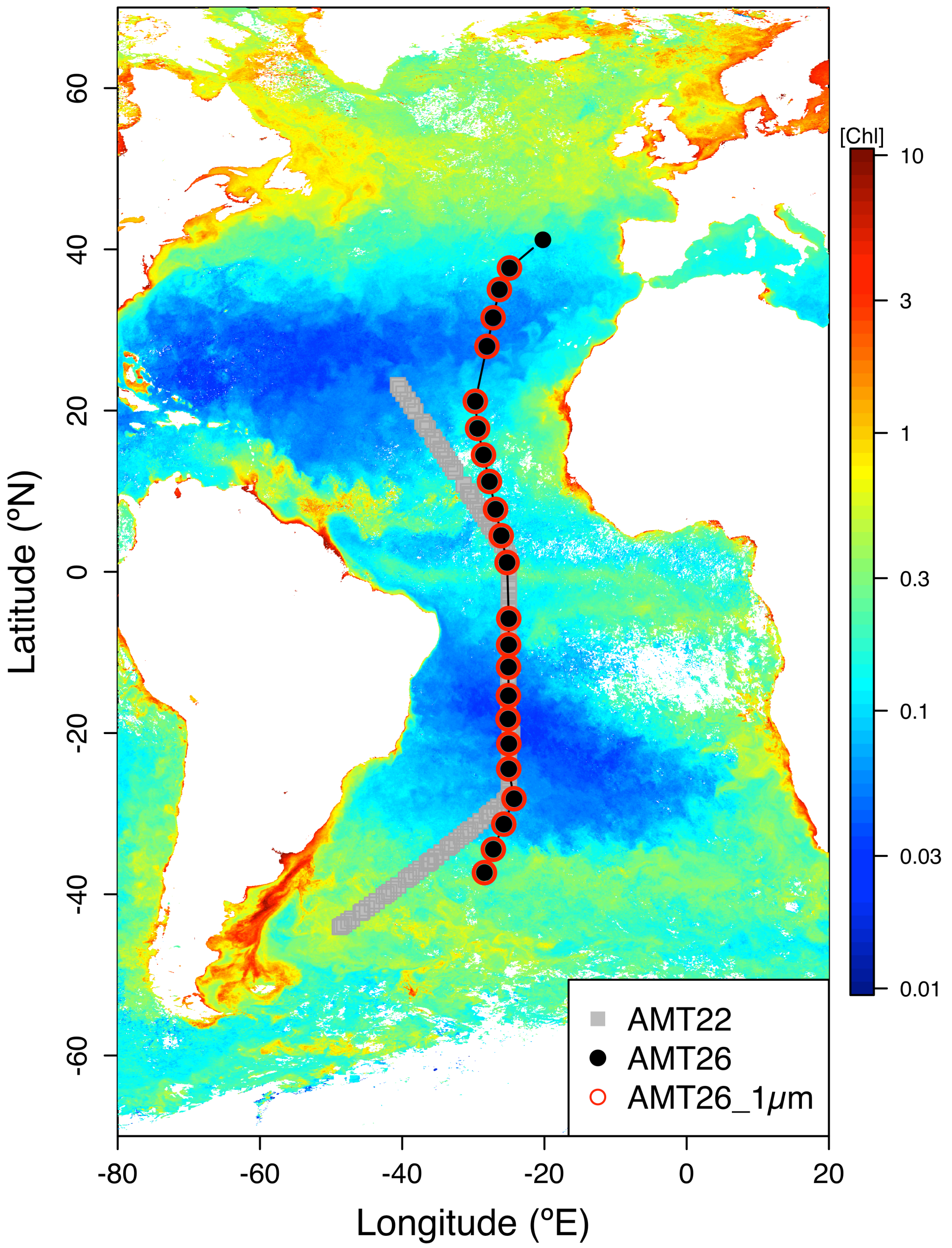

Supplementary Figure 1. Location of the stations sampled during the Atlantic Meridional Transect (AMT) cruises #22 and #26 superimposed onto the October 2016 Ocean Colour ESA Climate Change Initiative (v3.1) monthly Chlorophyll concentration ([Chl], units of mg m^{-3}) composite. The AMT26 stations (black circles) are used to check the homogeneous-sphere assumption and train the coated-sphere model of particles. AMT22 (grey squares) samples compose the validation dataset. AMT26_1μm (open red circles) indicates samples collected during the size-fractionation experiment. See methods for details and sampling strategies.

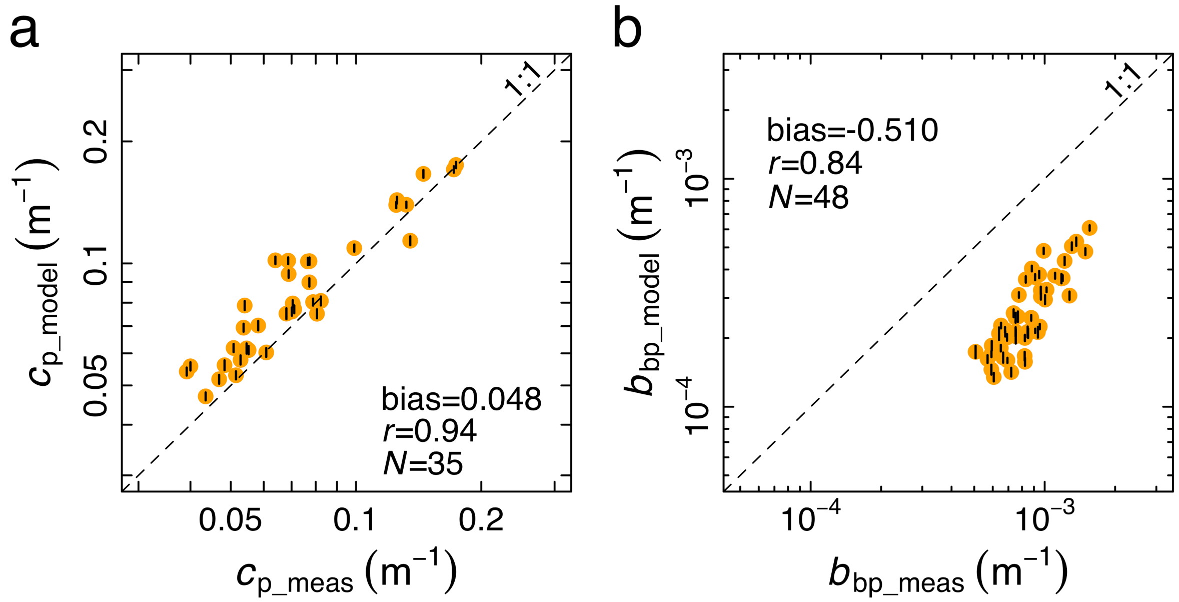

Supplementary Figure 2. Modelled vs. measured **(a)** particulate beam attenuation and **(b)** optical backscattering coefficients at 532 nm, $c_p(532)$ and $b_{bp}(532)$, for samples collected at the ocean surface (5 m) and DCM during the AMT26 cruise. Coefficients are modelled assuming particles as homogeneous spheres, with refractive index *n* equal to 1.06 for particles with diameters >1 µm and *n* equal to 1.11 for submicron particles. Changes in refractive index according to particle size do not allow to simultaneously reproduce beam attenuation and optical backscattering coefficients. This result is consistent with theoretical studies that have shown how the backscattering efficiency of submicron particles is insensitive to changes in the refractive index (see Figure 8 in Morel and Bricaud[1]). Systematic error (bias), Pearson's correlation coefficient (*r*) and number of observations (*N*) are shown. All *r* coefficients are statistically significant ($p<0.01$). Error bars represent the combined uncertainty (95% confidence intervals) as propagated from particle size distribution measurements (see methods).

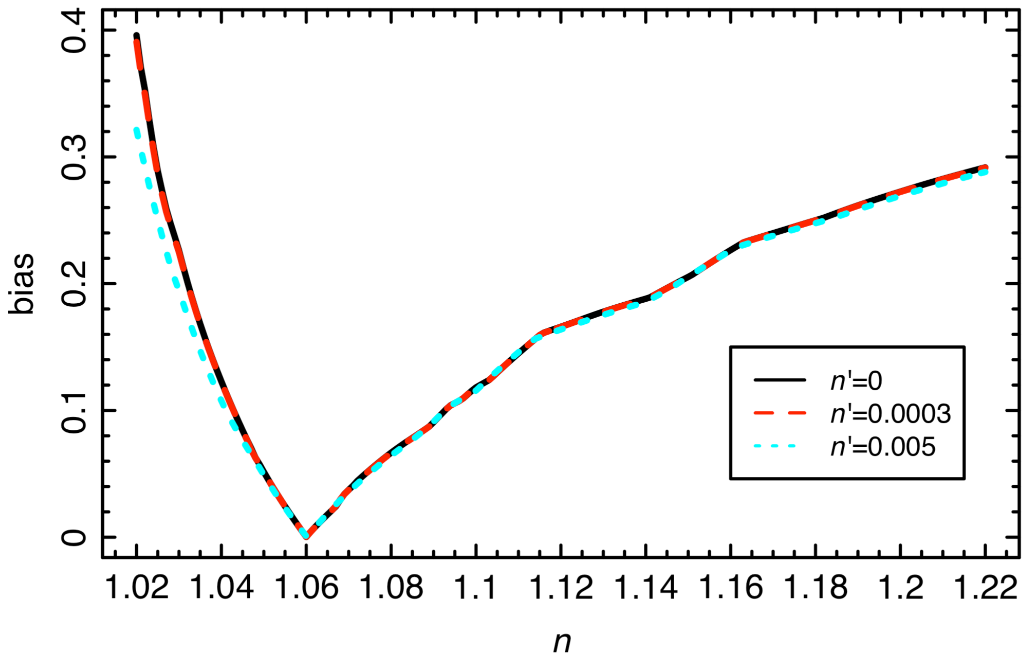

Supplementary Figure 3. Variations of the systematic error (bias in absolute value) between modelled (as homogeneous spheres) and measured $c_p(532)$ coefficients as a function of the real (n) and imaginary (n') parts of the complex refractive index[1] (m=n-in'). The n values vary between 1.02 and 1.22 in 0.001 increments. The null n' value indicates non-absorbing particles. n' values >0 are representative of the lowest and highest values measured around 530 nm for various phytoplankton species and heterotrophic nanoflagellates[2-4]. The analysis, made with surface (5 m) and DCM samples collected during the AMT26 cruise (N=35), reveals that the systematic error is minimized for n=1.06 regardless of variations in n'. Note that $c_p(532)$ coefficients are here modelled using the Anomalous Diffraction Approximation (ADA) according to Bricaud and Morel[5] and without correction of the acceptance angle effect[6], which explain the differences with respect to the values of bias for the various n in Supplementary Table 1.

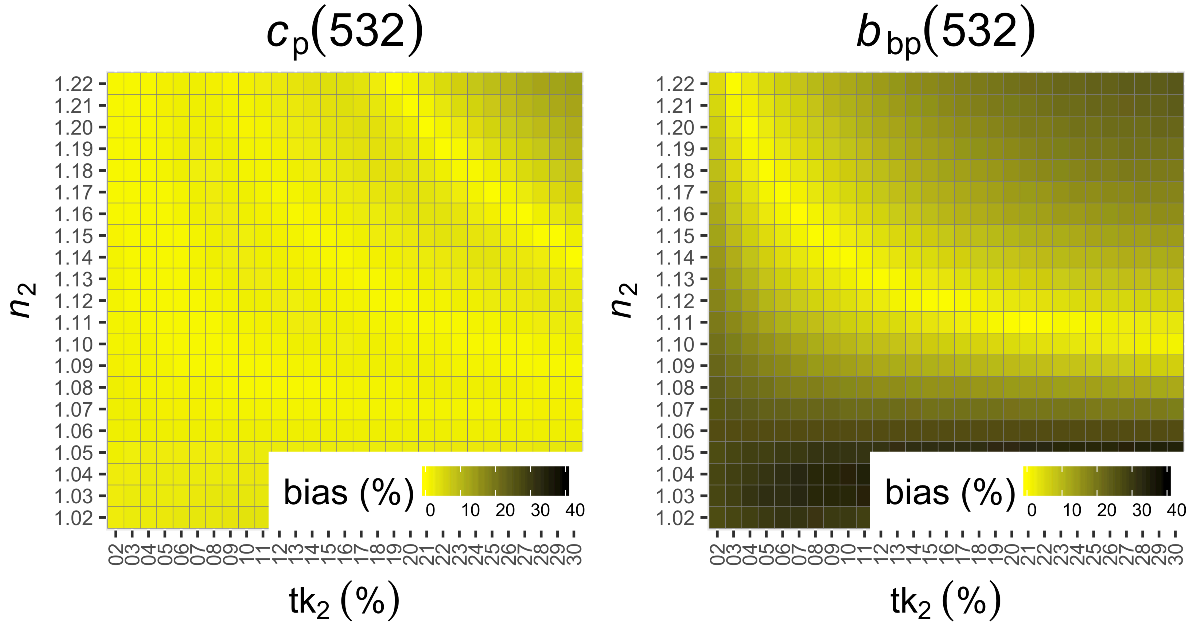

Supplementary Figure 4. Relative systematic error (bias, units of %) for comparisons of modelled vs. measured particulate beam attenuation ($c_p(532)$) and optical backscattering ($b_{bp}(532)$) coefficients at 532 nm, as a function of the coat thickness (tk_2) and the refractive index (n_2) of coated spheres. tk_2 is expressed as a percentage of the radius of the sphere. The analysis has been performed using samples collected at the ocean surface (5 m) and at the DCM during the AMT26 cruise. The equation to calculate the relative error is: $\text{bias} = 100 * \left|\text{median}\left((\log_{10}(\bar{x}_i) - \log_{10}(x_i))/\log_{10}(x_i)\right)\right|$ where $\bar{x}$ and x_i are the modelled and measured values, respectively.

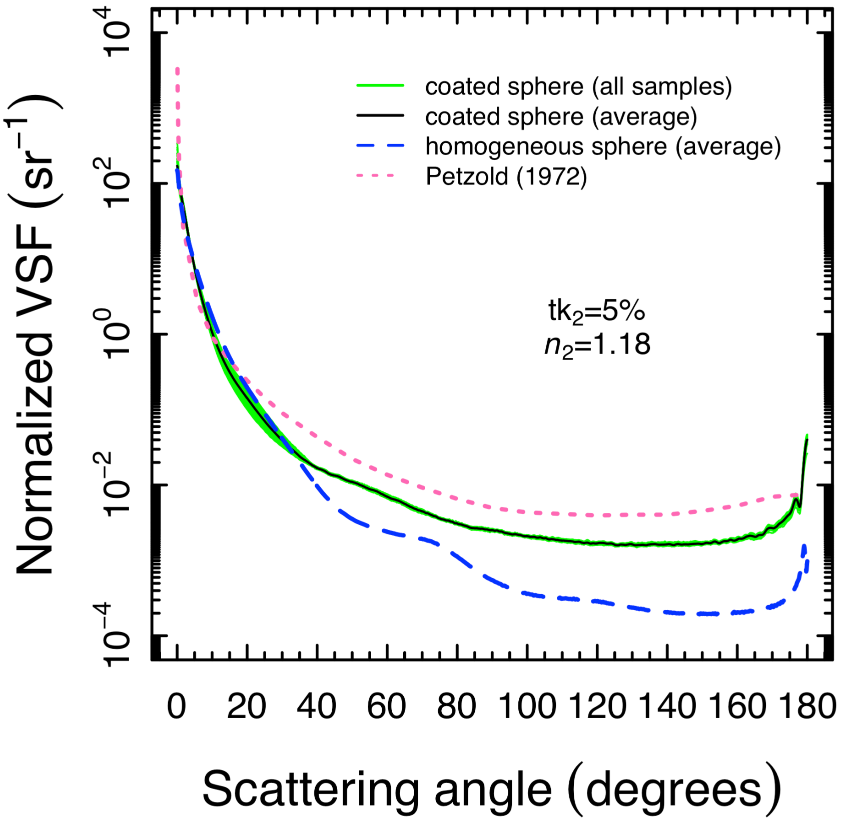

Supplementary Figure 5. Phase functions for the wavelength 532 nm (Normalized VSF; green solid lines) derived from particle size distributions (0.59-60 µm) measured at 5 m and at the DCM during the AMT26 cruise, by applying the coated-sphere model parameterisation with volume-averaged refractive index equal to 1.06, coat thickness (tk_2) and refractive index (n_2) equal to 5% and 1.18, respectively (see Eq. 10 in the main text). The average of all VSFs is shown (black solid line). The average of VSFs as obtained from the same particle size distributions and the homogeneous-sphere model (refractive index equal to 1.06) is shown for comparison (blue dashed line). The VSF measured by Petzold[7] at station 7 (09 July 1971), the Tongue of the Ocean, Bahama Islands, is shown as an example of in-situ clear waters (pink dotted line). The shapes of VSFs derived from the coated-sphere model are smooth and vary consistently with the measurement by Petzold[7] as a function of the scattering angle. When using the homogeneous-sphere model, the shape of VSF shows a peak around 70º and severely underestimated backward values (angles >90º).

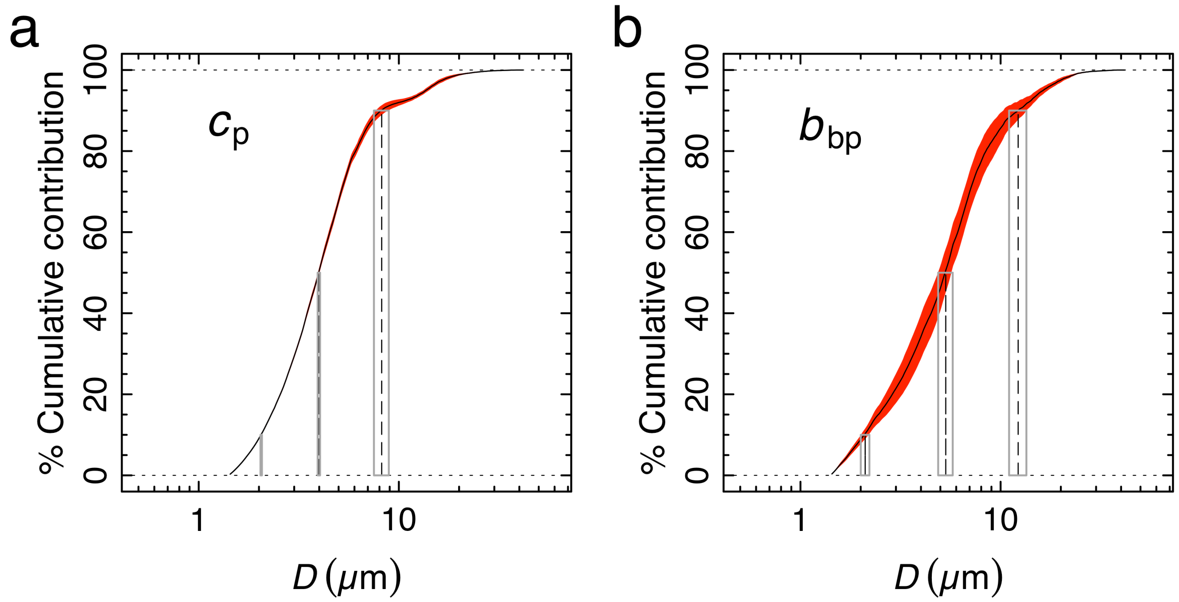

Supplementary Figure 6. Cumulative percent contributions (units of %) for modelled **(a)** particulate beam attenuation and **(b)** optical backscattering coefficients at 532 nm as a function of the particle diameter (*D*, units of μm) for samples collected during the AMT22 cruise. Black solid line indicates the median value and red areas the standard deviation obtained using the selected coated-sphere parameterisations. Vertical rectangles correspond to the median ± 1 standard deviation of the size thresholds of particle diameters causing the 10%, 50% and 90% of the signal, from left to right of each plot, respectively. Here, the contribution of submicron particles is equal to 0% because particle size distribution measurements during the AMT22 cruise were collected between 1.4-42 μm.

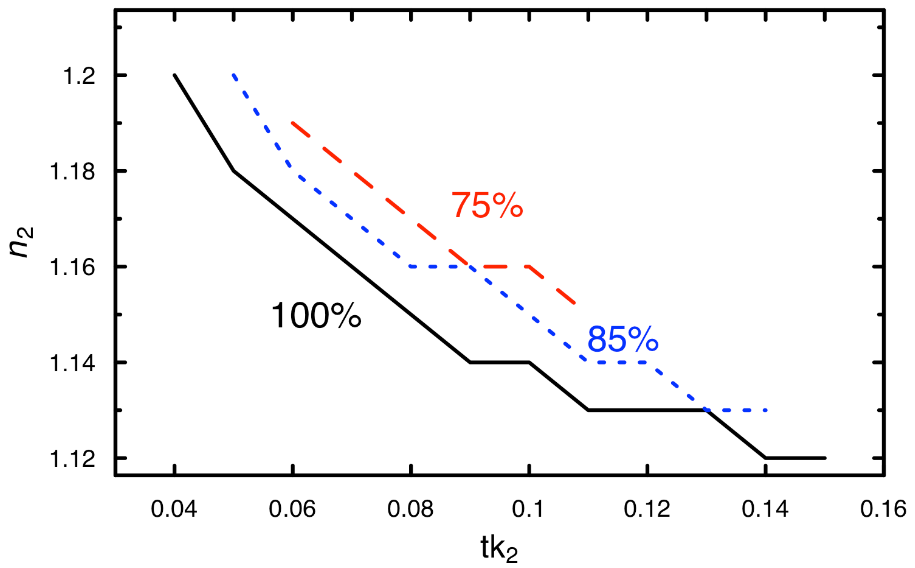

Supplementary Figure 7. Combinations of coat thickness (tk_2) and refractive index (n_2) that minimize the systematic error (bias) of predictions for $b_{bp}(532)$ coefficients using a coated-sphere model, with respect to in-situ optical measurements. Combinations are for varying proportions of coated (from 100% to 75%, with 5% steps) and homogeneous (from 0% to 25%, with 5% steps) particles, equally distributed from 0.588 to 60 µm of particle diameter. tk_2 is expressed as a fraction of the radius of the sphere. The volume-averaged refractive index (n) of coated and homogeneous spheres is set equal to 1.06. Simulations have been made using particle populations collected at the ocean surface (5 m) and the DCM level during the AMT26 cruise.

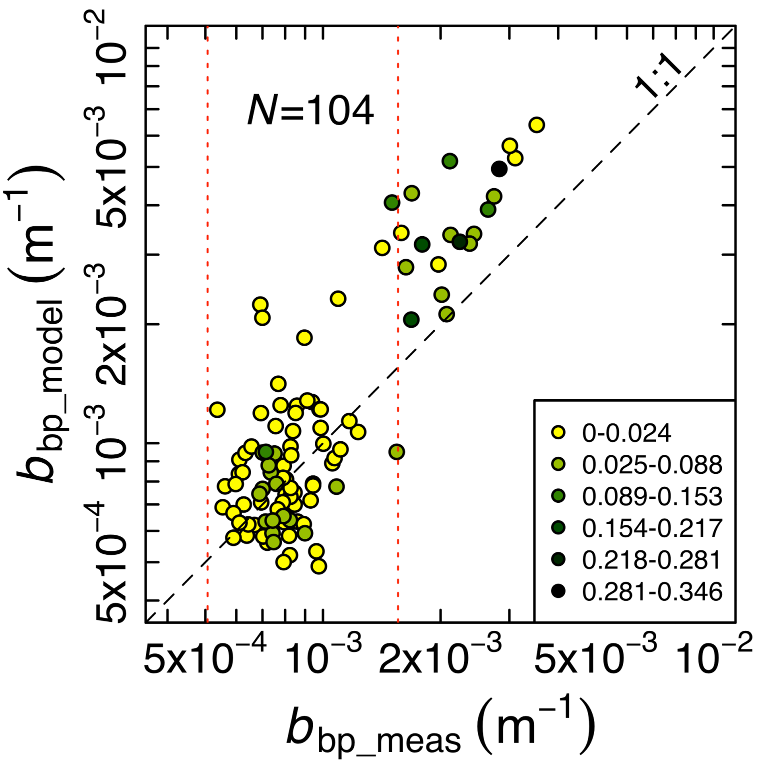

Supplementary Figure 8. Modelled vs. measured particulate optical backscattering coefficients at 532 nm for the AMT22 validation dataset (same as in Figure 4b). Coloured filled points indicate the fucoxanthin-to-total chlorophyll *a* ratio (dimensionless) for each sample as derived from high performance liquid chromatography pigment measurements following Van Heukelem and Thomas[8]. Sampling details are described in Tarran[9]. The fucoxanthin-to-total chlorophyll *a* pigment ratio is an estimator of the presence of diatoms in the community[10]. The average value of this ratio for samples with $b_{bp}(532)$ coefficients > 0.00156 m^{-1} is twice than for AMT22 samples with lower $b_{bp}(532)$ values, and about 2.4 times higher than $b_{bp}(532)$ surface values collected during the AMT26 cruise and used to parameterise the coated-sphere model. Red dotted lines indicate the range of measured values of the optical backscattering coefficients used to parameterise the coated-sphere model.

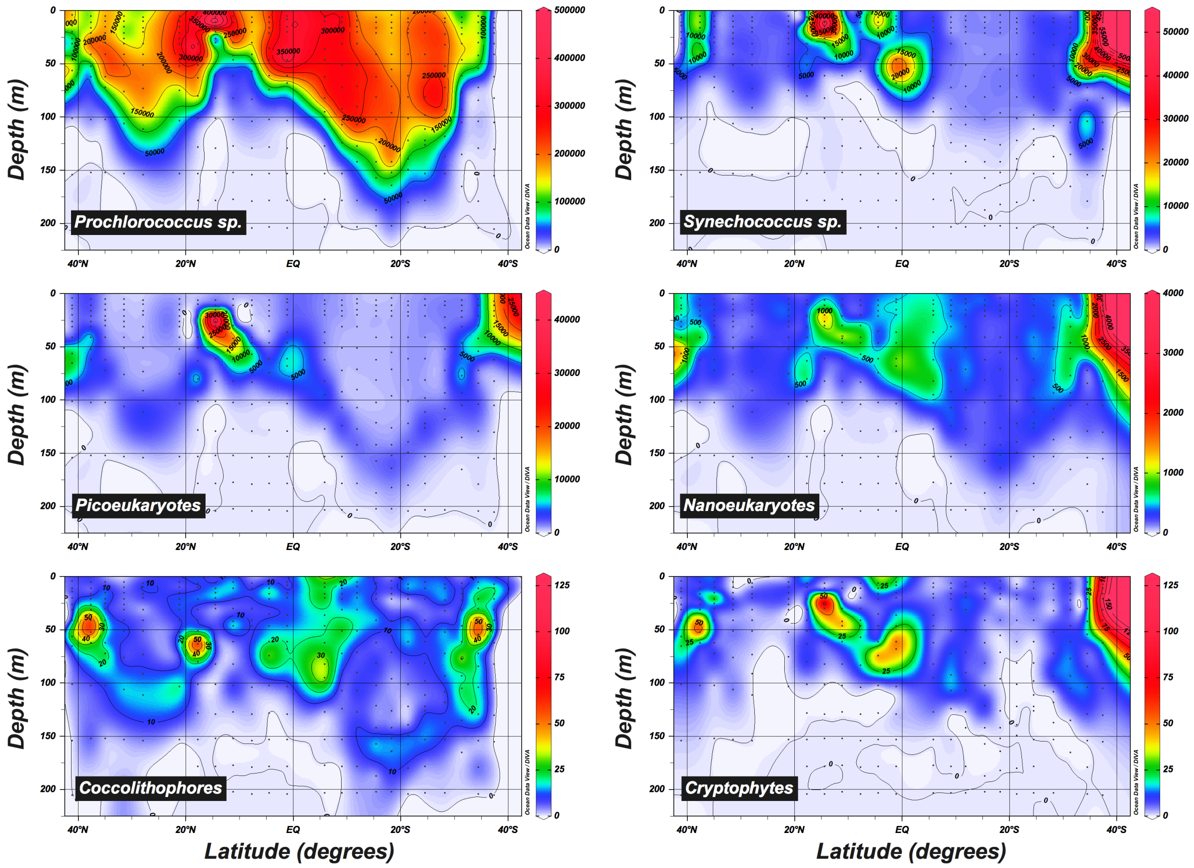

Supplementary Figure 9. Contour plots of phytoplankton abundance distribution (units of cells ml^{-1}) to 225 m during the AMT26 cruise as derived from flow cytometry analysis. Only the stations used to parameterise the coated-sphere model are shown. Dots indicate sampled depths. Detailed sampling strategy and analytical procedures used to enumerate phytoplankton are described in Rees[11] and Tarran et al.[12], respectively. Maps are drawn by the Ocean Data View software (R. Schlitzer, Ocean Data View, http://odv.awi.de).

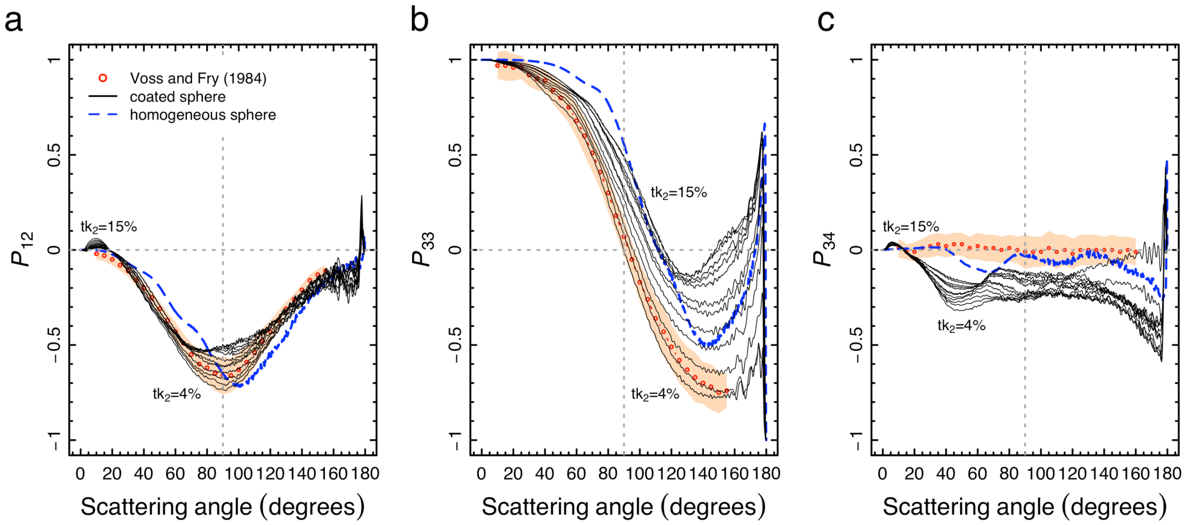

Supplementary Figure 10. Polarization components of the Mueller matrix as derived from the particle size distributions (0.59-60 µm) measured at 5 m and at the level of deep chlorophyll maximum during the AMT26 cruise (see Eq. 11 in the main text). The black solid lines represent the 12 selected coated-sphere parameterisations (see also Figure 3 in the main text) as derived from the average of all samples. The blue dashed line is obtained by assuming particles as homogeneous sphere and refractive index equal to 1.06. Red open circles and shaded areas are the average values and standard deviations, respectively, for a range of oceanic waters as reported in Tables 3 (for P_{34}) and 6 (for P_{12} and P_{33}) by Voss and Fry[13].

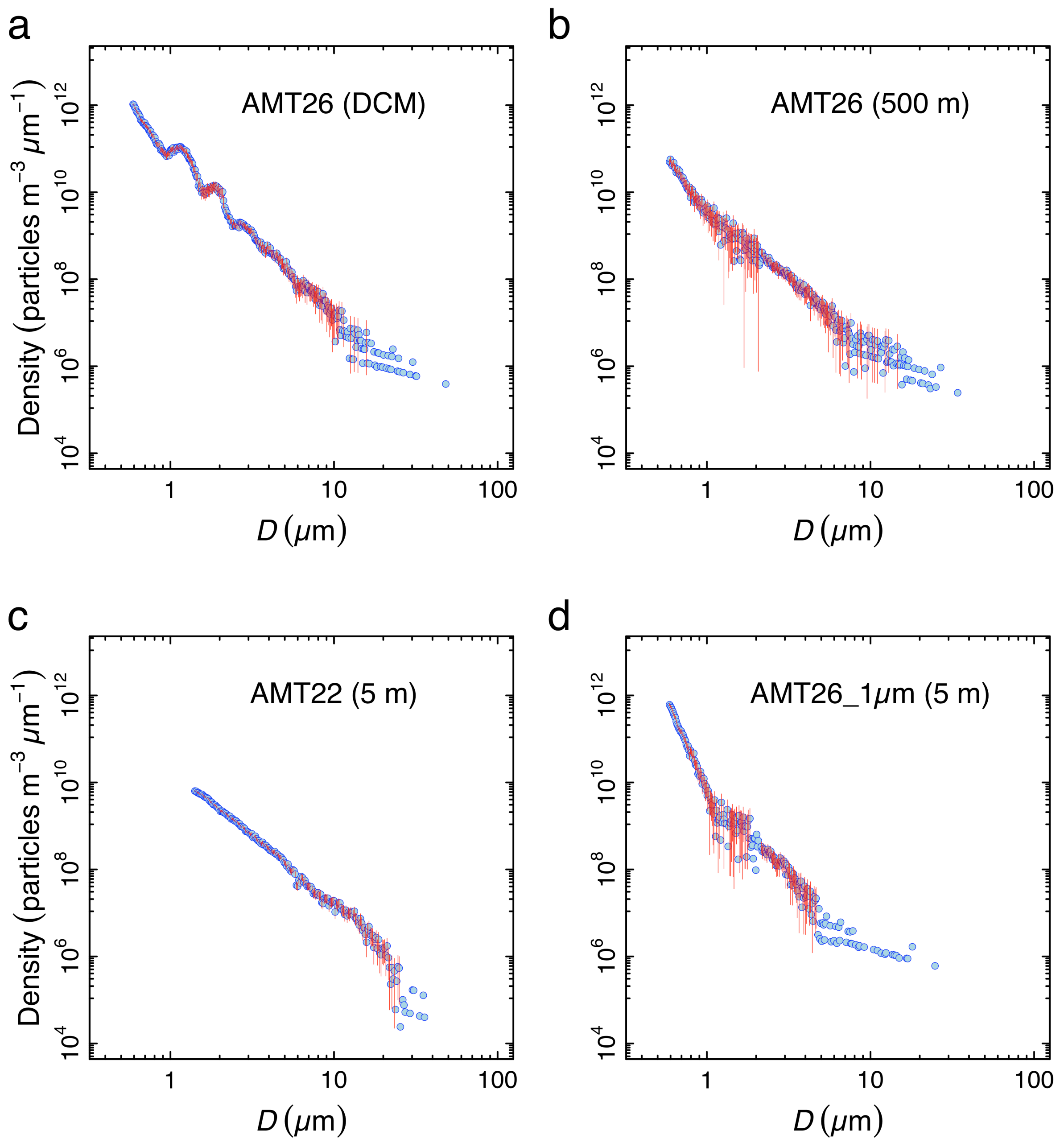

Supplementary Figure 11. Examples of particle size distributions (units of particles $m^{-3}\mu m^{-1}$) collected during the Atlantic Meridional Transects #22 and #26, as a function of the particle diameter (*D*, units of μm): **(a)** AMT26, 30 m, deep chlorophyll maximum (DCM), 14º30'N, 28º34'W; **(b)** AMT26, 500 m, 18º15'S, 25º07'W; **(c)** AMT22, 5 m, 19º02'S, 24º54'W; **(d)** AMT26_1μm, 5 m, 5º48'S, 25º01'W. Error bars represent the combined uncertainty (95% confidence intervals) as propagated from the summation of multiple particle size distribution measurements (see methods).

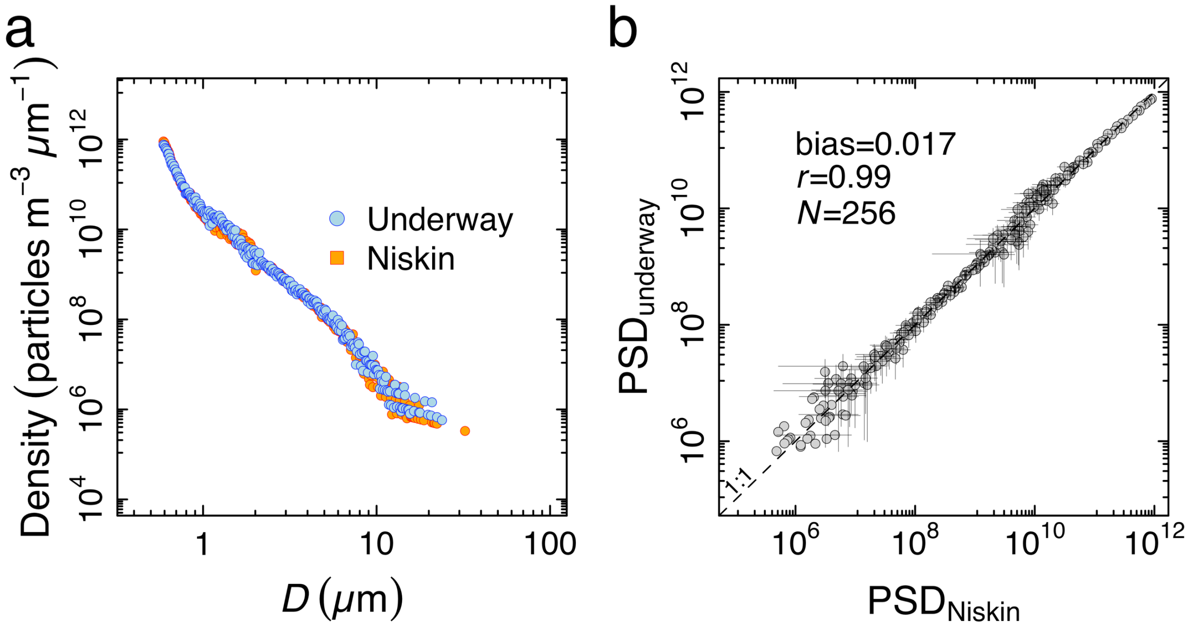

Supplementary Figure 12. (a) Particle size distributions (units of particles m^{-3} µm^{-1}) as a function of the particle diameter (*D*, units of µm), simultaneously collected from the ship's underway clean seawater supply before 1-µm filtration and from Niskin bottles at 5-m depth. Examples refer to samples collected during the AMT26 cruise at 5º48'S, 25º01'W (the same as Supplementary Fig. 11d). **(b)** Scatterplot of particle size distributions (PSD, units of particles m^{-3} µm^{-1}) shown in subplot **(a)**. Error bars represent the combined uncertainty (95% confidence intervals) as propagated from the summation of multiple particle size distribution measurements (see methods). Systematic error (bias), Pearson's correlation coefficient (*r*) and number of observations (*N*) are shown. The *r* coefficient is statistically significant (p<0.01).

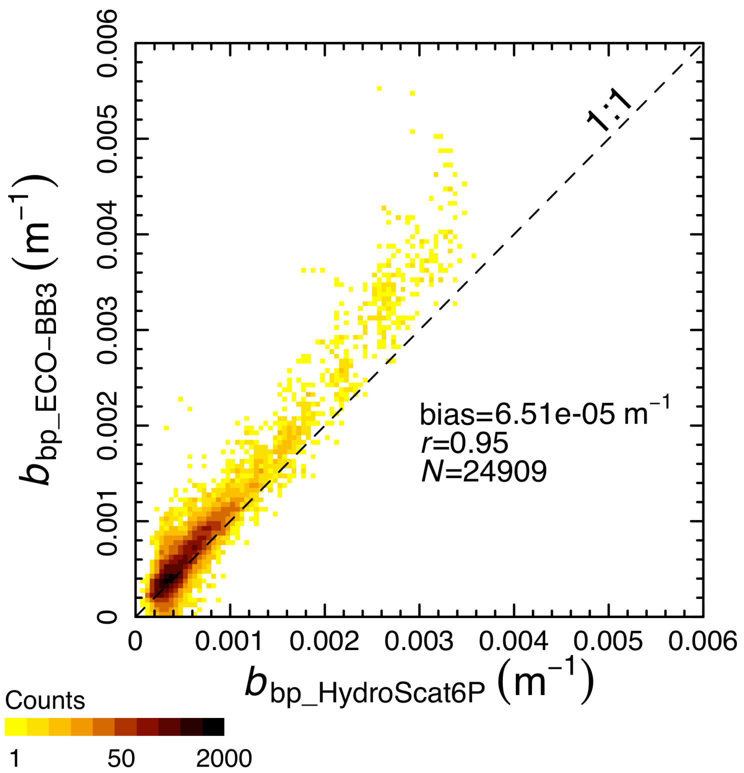

Supplementary Figure 13. Bivariate scatterplot comparing particulate optical backscattering coefficients (b_{bp}) as measured from a WETLabs ECO-BB3 (Seabird-Scientific) and a HydroScat-6P (HobiLabs) sensor, simultaneously collected with CTD casts along the AMT26 transect[11]. Comparison is shown for bands at 532 nm and 550 nm, for ECO-BB3 and HydroScat-6P respectively. See methods for protocols used to calculate $b_{bp}(532)$ for ECO-BB3 measurements. For upcast HydroScat-6P profiles, the angular scattering coefficients at 140° were converted into particulate angular scattering coefficient, $\beta_p(140, 550)$, by removing the contribution of pure seawater which in turn depends on water temperature, T, and salinity, S[14]. Measurements of T and S were taken from CTD casts[11]. $\beta_p(140, 550)$ was then converted to the particulate optical backscattering coefficient at 550 nm ($b_{bp}(550)$) using a χ factor equal to 1.14[15,16]. The $b_{bp}(550)$ vertical coefficients were smoothed with a moving-median filter (five-point window) and finally 1 m binned. The systematic error (bias) and the Pearson's correlation coefficient (r) show high consistency between optical measurements. The very small bias is due to differences between the wavelengths at which particulate optical backscattering has been measured. The r coefficient is statistically significant (p<0.01).

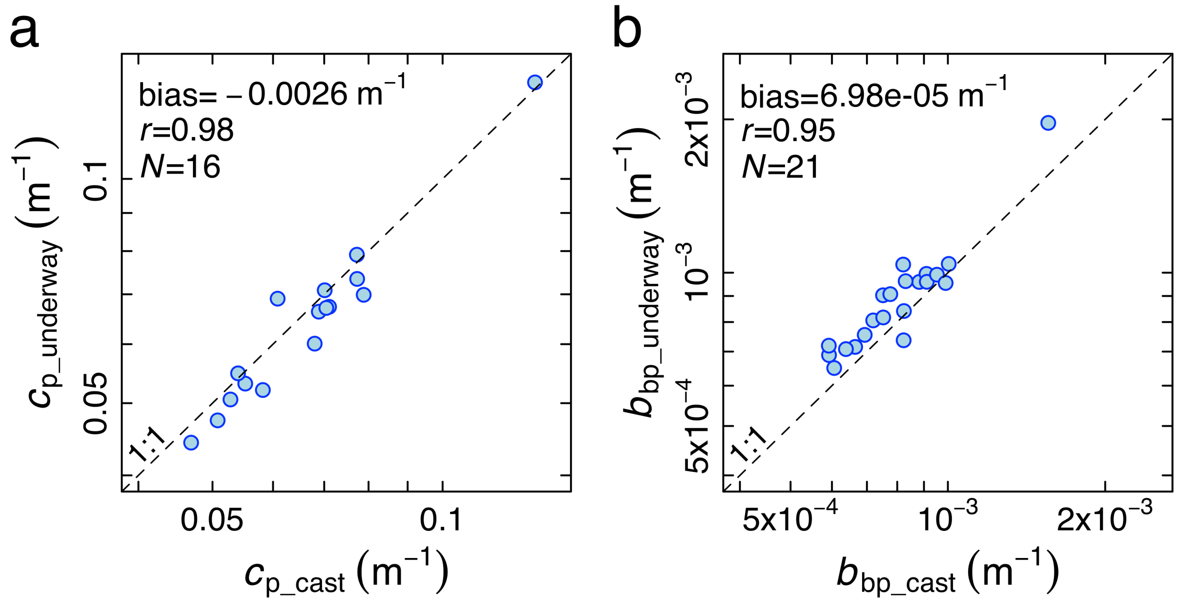

Supplementary Figure 14. (a) Particulate beam attenuation coefficients at 532 nm ($c_p(532)$) collected by a WETLabs ac-s sensor (Seabird-Scientific) from the ship's underway clean 5-m seawater supply compared to simultaneous independent measurements acquired with the WETLabs ac-9 sensor (Seabird-Scientific) at around 5 m during CTD casts. See methods for protocols. Comparisons are for samples collected before filtration through a 1 µm filter along the AMT26 transect (see methods). **(b)** as in **(a)** but for the particulate optical backscattering coefficient at 532 nm ($b_{bp}(532)$), as measured by two WETLabs ECO-BB3 sensors (Seabird-Scientific). See methods for protocols. In each plot, the systematic error (bias) and the Pearson's correlation coefficient (r) show high consistency between optical measurements collected by using the ship's underway system and those simultaneously collected during CTD casts. The bias values shown in plot **(a)** and **(b)** were used to adjust to the best underway optical measurements after water filtration through a 1 µm filter and remove any under-corrected optical effect such as the wall chamber for $b_{bp}(532)$. All r coefficients are statistically significant ($p<0.01$).

Section 2

Supplementary Table 1. Systematic error (bias) and the Pearson's correlation coefficient (r) of match-up analyses between modelled with homogeneous spheres (i.e., Mie theory) and in-situ measured $c_p(532)$ and $b_{bp}(532)$ coefficients, as a function of the refractive index n. Mie simulations are executed using the scattnlay software[17]. Number of observations (N) and the pvalue (p) of Pearson's correlations as derived from a two-tailed Student's t-test (confidence level equal to 99%; N-2 degrees of freedom) are reported. All r values are statistically significant. The bold-highlighted values indicate the lowest bias for $c_p(532)$ and $b_{bp}(532)$ comparisons.

	$c_p(532)$				$b_{bp}(532)$			
n	**bias**	***r***	***p***	***N***	**bias**	***r***	***p***	***N***
1.02	-0.494	0.93	1.9e-15	35	-2.024	0.86	3.2e-15	48
1.03	-0.284	0.93	1.6e-15	35	-1.636	0.86	6.2e-15	48
1.04	-0.165	0.93	7.1e-16	35	-1.325	0.85	2.9e-14	48
1.05	-0.080	0.93	2.1e-16	35	-1.047	0.84	9.9e-14	48
1.06	**-0.018**	0.94	5.9e-17	35	-0.821	0.83	2.3e-13	48
1.07	0.024	0.94	2.4e-17	35	-0.624	0.83	3.7e-13	48
1.08	0.063	0.94	1.8e-17	35	-0.438	0.82	7.6e-13	48
1.09	0.092	0.94	2.3e-17	35	-0.280	0.81	2.0e-12	48
1.10	0.121	0.94	4.4e-17	35	-0.134	0.81	4.6e-12	48
1.11	0.150	0.94	1.1e-16	35	**-0.005**	0.80	6.4e-12	48
1.12	0.175	0.93	2.7e-16	35	0.102	0.80	7.2e-12	48
1.13	0.189	0.93	6.8e-16	35	0.193	0.80	6.1e-12	48
1.14	0.207	0.93	1.6e-15	35	0.270	0.81	3.9e-12	48
1.15	0.227	0.92	3.6e-15	35	0.340	0.81	2.1e-12	48
1.16	0.247	0.92	7.5e-15	35	0.407	0.82	1.4e-12	48
1.17	0.267	0.91	1.5e-14	35	0.457	0.83	5.0e-13	48
1.18	0.280	0.91	3.1e-14	35	0.507	0.83	2.6e-13	48
1.19	0.295	0.91	6.0e-14	35	0.557	0.84	1.3e-13	48
1.20	0.308	0.90	1.2e-13	35	0.598	0.84	5.5e-14	48
1.21	0.320	0.90	2.3e-13	35	0.641	0.85	2.8e-14	48
1.22	0.332	0.89	4.5e-13	35	0.680	0.85	1.3e-14	48

Section 3

Supplementary References

1. Morel, A. & Bricaud, A. Inherent optical properties of algal cells including picoplankton: Theoretical and experimental results. *Canadian Bulletin of Fisheries and Aquatic Sciences* **214**, 521–559 (1986).
2. Morel, A. & Ahn, Y.-H. Optics of heterotrophic nanoflagellates and ciliates - a tentative assessment of their scattering role in oceanic waters compared to those of bacterial and algal cells. *J. Mar. Res.* **49**, 177–202 (1991).
3. Stramski, D. & Kiefer, D. Light scattering by microorganisms in the open ocean. *Prog. Oceanogr.* **28**, 343–383 (1991).
4. Ahn, Y.-H., Bricaud, A. & Morel, A. Light backscattering efficiency and related properties of some phytoplankters. *Deep-Sea Res.* **39**, 1835–1855 (1992).
5. Bricaud, A. & Morel, A. Light attenuation and scattering by phytoplankton cells: a theoretical modeling. *Appl. Optics* **25**, 571–580 (1986).
6. Boss, E., Slade, W., Behrenfeld, M. & Dall'Olmo, G. Acceptance angle effects on the beam attenuation in the ocean. *Opt. Express* **17**, 1535–1550 (2009).
7. Petzold, T. J. *Volume scattering functions for selected ocean waters. Final Technical Report No N62269-71-C-0676* (University of California San Diego, 1972).
8. Van Heukelem, L. & Thomas, C. S. Computer-assisted high-performance liquid chromatography method development with applications to the isolation and analysis of phytoplankton pigments. *J. Chromatogr. A* **910**, 31–49, doi: 10.1016/S0378-4347(00)00603-4 (2001).
9. Tarran, G. *AMT22 cruise report* (http://www.amt-uk.org, 2012).
10. Claustre, H. The trophic status of various oceanic provinces as revealed by phytoplankton pigment signatures. *Limnol. Oceanogr.* **39**, 1206–1210 (1994).
11. Rees, A. *AMT26 cruise report* (http://www.amt-uk.org, 2016).
12. Tarran, G. A., Heywood, J. L. & Zubkov, M. V. Latitudinal changes in the standing stocks of nano- and picoeukaryotic phytoplankton in the Atlantic Ocean. *Deep-Sea Res. Pt. II* **53**, 1516–1529 (2006).
13. Voss, K. J. & Fry, E. S. Measurement of the Mueller matrix for ocean water. *Appl. Optics* **23**, 4427–4439 (1984).
14. Zhang, X., Hu, L. & He, M.-X. Scattering by pure seawater: Effect of salinity. *Opt. Express* **17**, 5698–5710 (2009).
15. Stramski, D., Reynolds, R. A., Babin, M., Kaczmarek, S., Lewis, M. R., Rottgers, R., Sciandra, A., Stramska, M., Twardowski, M. S., Franz, B. A. & Claustre, H. Relationships between the surface concentration of particulate organic carbon and optical properties in the eastern South Pacific and eastern Atlantic Oceans. *Biogeosci.* **5**, 171–201 (2008).
16. Hobilabs. *HydroScat-6P, spectral backscattering sensor & fluorometer* (www.hobilabs.com, 2010).
17. Peña, O. & Pal, U. Scattering of electromagnetic radiation by a multilayered sphere. *Computer Physics Communications* **180**, 2348–2354 (2009).
